# A Case Report: A 75-Year-Old Male With Abdominal Pain, Diarrhea, and Hypotension

**DOI:** 10.7759/cureus.29166

**Published:** 2022-09-14

**Authors:** Marcia Machado, Daniela Neto, David Paiva, Carlos Fernandes, Jorge Cotter

**Affiliations:** 1 Internal Medicine, Hospital Senhora da Oliveira Guimarães, Guimarães, PRT; 2 Internal Medicine, Hospital Senhora Da Oliveira Guimaraes, Guimarães, PRT

**Keywords:** acute abdominal pain, emergency service, hemorrhagic shock, shock, abdominal aortic rupture

## Abstract

The differential diagnosis of abdominal pain ranges from benign to life-threatening conditions. This case report describes the importance of the differential diagnosis and a faster and more accurate diagnosis. A 75-year-old male presented to the emergency room (ER) with diffuse abdominal pain, associated nausea, vomiting, diarrhea, and a fever of 38.5ºC since the previous day. Medical history included hypertension, dyslipidemia, and obesity. Clinical examination showed hypotension and a distended abdomen with diffuse tenderness in all quadrants. Blood tests revealed a hemoglobin of 11.3 g/dL, and an arterial blood gas test revealed metabolic acidosis and lactate of 8 mmol/L. Contrast-enhanced computed tomography (CT) of the abdomen and pelvis revealed a large aneurysm in the infrarenal aorta with an extension of about 17x8x8 cm and an exuberant mural thrombus. The patient underwent endovascular treatment of the aneurysm; however, he died during surgery.

## Introduction

The definition of an abdominal aortic aneurysm (AAA) is based on the diameter of the abdominal aorta; an abdominal aortic diameter of 3.0 cm or more is considered to be aneurysmal [1] and causes 1-3% of all deaths among men aged 65-85 years in developed countries [2]. AAA is typically asymptomatic until a catastrophic rupture event. If symptomatic, the clinical triad is abdominal or back pain of sudden onset, hypotension, and a pulsatile mass [3] although only 25-50% of patients demonstrate all signs. The differential diagnoses include acute myocardial infarction, kidney stones, and gastrointestinal diseases such as perforated ulcers [2]. The most important risk factors are smoking, male sex, atherosclerosis, and positive family history [4].

## Case presentation

A 75-year-old male presented to the ER with diffuse abdominal pain, associated nausea, vomiting, diarrhea, and a fever of 38.5ºC within one day of evolution. The past medical history of the patient was remarkable for long-standing hypertension, dyslipidemia, and obesity. He did not have the habit of smoking or alcohol consumption. His medication included amlodipine, atorvastatin, and acetylsalicylic acid. Upon examination, the patient was alert and looked distressed while his pulse rate was 130 bpm, blood pressure was 67/38 mmHg, respiratory rate was 26 bpm, and temperature was 36.7℃. His abdomen was distended, and he had diffuse tenderness on palpation of the abdomen and no other remarkable findings. Bowel sounds were present and normal. His peripheral pulses were reduced and capillary refill time was increased. Initial consideration included sepsis; this patient was resuscitated with 0.9% NaCl at 30 ml/kg and broad-spectrum antibiotics. Laboratory investigation revealed a hemoglobin level of 11.3 g/dL, creatinine 2,78 mg/dL, and urea 70 mg/dL. Arterial blood gas revealed metabolic acidosis and lactate of 8 mmol/L (Table 1).

**Table 1:** Blood Test Results

Blood test	Results	Normal range
White blood cells	25.6	4.5 - 11x10^3^/µL
Hemoglobin	11.3	13 - 17.0 g/dL
Platelet count	201	130 - 400x10^3^/µL
Blood urea nitrogen	32.67	8.4 - 25.7 mg/dL
Creatinine	2.78	0.72 - 1.25 mg/dL
Total bilirubin	1.0	0.2 - 1.2 mg/dL
Aspartate transaminase	19	5 - 34 U/L
Alanine transaminase	15	10 - 55 U/L
Alkaline phosphatase	73	40 - 150 U/L
Amylase	55	40 – 140 U/L
Lipase	146	10 – 140 U/L
Arterial blood gas		
pH	7.351	7.351 - 7.45
pCO2	28.1	35 - 45 mmHg
pO2	75.7	80.0-100.0 mmHg
HCO3	15.2	22.0-31.0 mmol/L
Lactate	8	0.5-2.2 mmol/L

**Figure 1:**
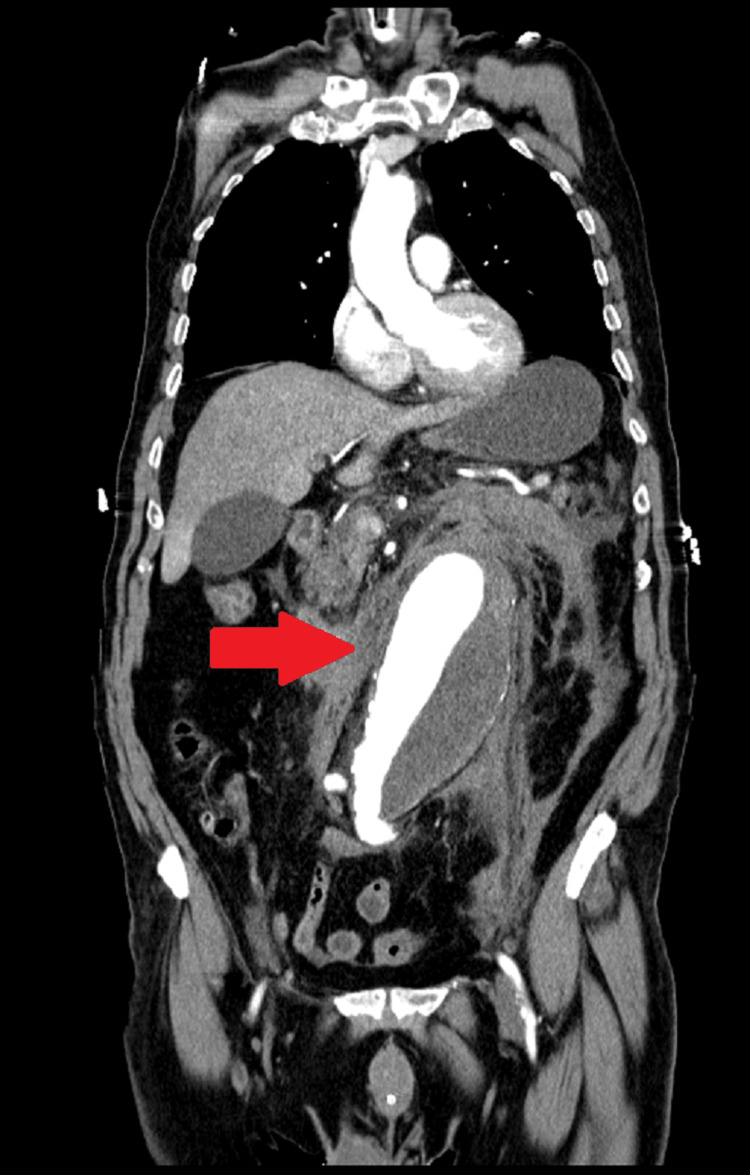
CT scan of the abdomen and pelvis with an aortic abdominal aneurysm and thrombus (arrow) CT: computed tomography

Point-of-care ultrasound was not available. Contrast-enhanced computed tomography (CT) of the abdomen and pelvis was performed and revealed a large aneurysm in the infrarenal aorta with a longitudinal extension of about 17 cm, starting distally to the emergence of the renal arteries until the bifurcation of the iliac arteries and with slight extension to the proximal left iliac artery, of 8.1 x 8.0 cm, and did not exclude aneurysmal rupture or dissection. A mural thrombus about 4.6 cm thick was present (Figure 1).

The patient remained hypotensive despite the large volume of fluid resuscitation, and a jugular central venous catheter (CVC) was placed in order to start treatment with norepinephrine and referred to a vascular surgery team because there was a possibility that the aneurysm was ruptured. The patient was admitted for surgical treatment. During surgery, it was possible to observe a ruptured aneurysm, aortic thrombosis, and intestinal ischemia. However, it was not possible to repair the aneurysm, and the patient progressed to refractory hemorrhagic shock and died during the surgery.

## Discussion

Getting the correct diagnosis quickly can be difficult and challenging. The diagnosis is made by CT scan with angiography, ultrasound, or magnetic resonance angiography, however, a CT scan with angiography is the imaging modality of choice for the diagnosis and follow-up [5]. Bedside ultrasound is an exam that allows rapid evaluation and differential diagnosis. It is useful to identify AAA with 99% sensitivity and 98% specificity and can help establish a diagnosis and treatment more quickly [6]. In the case reported, abdominal ultrasound at the bedside was not performed and could have contributed to this outcome. It is important that the ultrasound is available in the emergency room and that the emergency physician is able to perform an ultrasound at the patient's bedside. Endovascular aneurysm repair (EVAR) or open surgical repair (OSR) is the currently available treatment for ruptured aneurysms and the decision should be made on a case-by-case basis. Perioperative mortality in patients with a ruptured abdominal aortic aneurysm was 15-44% for EVAR and 31-48% for OSR. Medical professionals must be vigilant for early complications such as pulmonary (42%) or cardiac (18%) decompensation, colon ischemia (9%), and acute kidney injury (17%) [1].

## Conclusions

The evaluation of abdominal pain requires an extensive differential diagnosis, including acute aortic syndrome, such as a ruptured aneurysm, which quickly becomes life-threatening and requires prompt medical treatment. Despite CT scan with angiography remaining the imaging modality of choice, bedside ultrasound helps establish a correct diagnosis and rule out other causes.
